# Diagnostic and Prognostic Value of SCUBE-1 in COVID-19 Patients

**DOI:** 10.5811/westjem.18586

**Published:** 2024-10-02

**Authors:** Vildan Ozer, Ozgen Gonenc Cekic, Ozlem Bulbul, Davut Aydın, Eser Bulut, Firdevs Aksoy, Mehtap Pehlivanlar Kucuk, Suleyman Caner Karahan, Ebru Emel Sozen, Esra Ozkaya, Polat Kosucu, Yunus Karaca, Suleyman Turedi

**Affiliations:** * Karadeniz Technical University, School of Medicine, Department of Emergency Medicine, Trabzon, Türkiye; † SBU Kanuni Training and Research Hospital, Department of Emergency Medicine, Trabzon, Türkiye; ‡ SBU Kanuni Training and Research Hospital, Department of Chest Diseases, Division of Intensive Care Medicine, Trabzon, Türkiye; § SBU Kanuni Training and Research Hospital, Department of Radiology, Trabzon, Türkiye; ∥ Karadeniz Technical University, School of Medicine, Department of Infectious Diseases and Clinical Microbiology, Trabzon, Türkiye; ¶ Karadeniz Technical University, School of Medicine, Department of Chest Diseases, Division of Intensive Care Medicine, Trabzon, Türkiye; # Karadeniz Technical University, School of Medicine, Department of Biochemistry, Trabzon, Türkiye; ** SBU Kanuni Training and Research Hospital, Department of Infectious Diseases and Clinical Microbiology, Trabzon, Türkiye; †† Karadeniz Technical University, School of Medicine, Department of Medical Microbiology, Trabzon, Türkiye; ‡‡ Karadeniz Technical University, School of Medicine, Department of Radiology, Trabzon, Türkiye

## Abstract

**Introduction:**

The workload of physicians increased due to the number of patients presenting with suspicion of coronavirus 2019 (COVID-19) and the prolonged wait times in the emergency department during the COVID-19 pandemic. Signal peptide-CUB-EGF domain-containing protein 1 (SCUBE-1) is a protein present in platelets and endothelial cells; it is activated by inflammation from COVID-19 and may be associated with COVID-19’s known thrombotic risk. We aimed to determine whether SCUBE-1 levels are diagnostically correlated in suspected COVID-19 patients, and whether SCUBE-1 correlated with severity of disease and, therefore, might be useful to guide hospitalization/discharge decisions.

**Methods:**

The suspected COVID-19 patients cared for at tertiary healthcare institutions for one year between May 2021–May 2022 were examined in this study. The subjects were both suspected COVID-19 patients not ultimately found to have COVID-19 and those who were diagnosed with COVID-19. By modifying the disease severity scoring systems present in COVID-19 guidelines in 2021, the COVID-19-positive patient group was classified as mild, moderate, severe, and critical, and compared using the SCUBE-1 levels. Moreover, SCUBE-1 levels were compared between the COVID-19 positive group and the COVID-19 negative group.

**Results:**

A total of 507 patients were considered for the present study. After excluding 175 patients for incomplete data and alternate comorbid organ failure. we report on 332 patients (65.5%). Of these 332 patients, 80 (24.0%) were COVID-19 negative, and 252 (76.0%) were COVID-19 positive. Of 252 (100%) patients diagnosed with COVID-19, 74 (29.4%) were classified as mild, 95 (37.7%) moderate, 45 (17.8%) severe, and 38 (15.1%) critical. The SCUBE-1 levels were statistically different between COVID-19 positive (8.48 ± 7.42 nanograms per milliliter [ng/mL]) and COVID-19 negative (1.86 ± 0.92 ng/mL) patients (*P* < 0.001). In the COVID-19 positive group, SCUBE-1 levels increased with disease severity (mild = 3.20 ± 1.65 ng/mL, moderate = 4.78 ± 2.26 ng/mL, severe = 13.68 ± 3.95 ng/mL, and critical = 21.87 ± 5.39 ng/mL) (*P* < 0.001). The initial SCUBE-1 levels of discharged patients were significantly lower than those requiring hospitalization (discharged = 2.89 ng/mL [0.55–8.60 ng/mL]; ward admitted = 7.13 ng/mL [1.38–21.29 ng/mL], and ICU admitted = 21.19 ng/mL [10.58–37.86 ng/mL]) (*P* < 0.001).

**Conclusion:**

The SCUBE-1 levels were found to be differentiated between patients with and without COVID-19 and to be correlated with the severity of illness.

Population Health Research Capsule What do we already know about this issue?

*During the pandemic, the increase in the length of time that patients spent in the ED and the resultant crowding led to higher mortality.*

What was the research question?

*Can SCUBE-1 levels serve as a diagnostic marker in COVID-19 and be correlated with the severity of the disease?*

What was the major finding of the study?

*SCUBE-1 levels were higher in COVID-19 positive patients (P < 0.001) and increased with disease severity (P < 0.001).*

How does this improve population health?

*Using SCUBE-1 as a biomarker enables timely diagnosis of COVID-19 and severity assessment when RT-PCR results are delayed.*

## INTRODUCTION

The coronavirus 2019 (COVID-19) pandemic has had a huge impact^1^ ^,^ ^2^ with more than 770 million confirmed cases and more than 6.9 million deaths reported worldwide as of September 2023.^3^ Although hospital and emergency department (ED) admission rates decreased in the first period of the COVID-19 pandemic, these rates returned to pre-pandemic levels over time. Previous studies showed that the duration of time spent by patients in the ED increased during the pandemic period.^4^ ^,^ ^5^ This led to ED crowding, which in turn contributed to increased inhospital mortality.^6^ For this reason, it would be useful to determine which patients can be managed as outpatients, and which need admission and to what level of care. It is also crucial to promptly diagnose and provide treatment for this patient group to manage disease-related prognosis because the mortality rate of patients who visit hospitals with COVID-19 and require intensive care admission is high.^7^

As a member of the signal peptide-CUB-epidermal growth factor domain-containing protein (SCUBE) gene family, SCUBE-1 is a cell surface glycoprotein predominantly located in platelets and, to a lesser extent, in endothelial cells. The SCUBE-1 is stored in the alpha granules of inactive platelets and migrates to the platelet surface after activation by thrombin and released as small soluble particles that are incorporated into the thrombus. Unlike other members of the SCUBE gene family, SCUBE-1 tends to cause inflammation and thrombosis and can be evaluated as a prognostic factor in platelet activation and thrombotic diseases.^8^ ^,^ ^9^ Infection with COVID-19 predisposes patients to venous and arterial thromboembolisms due to excessive inflammation, hypoxia, immobilization, and disseminated intravascular coagulation.^10^ Previous studies have shown that the thrombotic complication rate increases with increasing severity of the disease.^1^ ^,^ ^2^ ^,^ ^11^ ^,^ ^12^

We aimed to determine whether SCUBE-1 levels are diagnostically correlated in suspected COVID-19 cases and to determine whether SCUBE-1 correlated with severity of disease and, therefore, might be useful to guide hospitalization/discharge decisions.

## METHODS

### Study Design

The study had a prospective and observational cohort design and recruited patients with suspected COVID-19 who visited tertiary healthcare institutions that managed patients with COVID-19, for one year between May 2021–May 2022. The patients in the COVID-19 (+) group were suspected of having COVID-19 and were diagnosed with COVID-19 by laboratory tests, imaging, and real-time polymerase chain reaction (RT-PCR) testing. The COVID-19 (−) group were those patients who had symptoms but did not have COVID-19 and were discharged. Patient exclusion criteria were as follows: incomplete data records; lack of consent; aged <18 years; and conditions that may alter SCUBE-1 levels due to predisposition to thrombosis, including pregnancy, acute renal failure, acute myocardial infarction, acute ischemic cerebrovascular disease, acute mesenteric ischemia at the time of diagnosis, peripheral arterial disease, liver failure, heart failure, or malignancy.

The disease severity of patients in the COVID-19 (+) group was determined in June 2020 using the classification introduced in guidelines published by the US National Center for Immunization and Respiratory Diseases, Division of Viral Diseases.^13^ Using these guidelines, patients are classified as, 1-mild to moderate, 2-severe, or 3-critical. To determine whether there was a difference between “mild” and “moderate,” patients in the “mild to moderate” group based on the SCUBE-1 level were classified further as “mild” vs “moderate” in July 2023 by following the “COVID-19 Treatment Guide” published by the US National Institutes of Health.^14^

As our goal in this study was to determine whether there were differences in the SCUBE-1 level, based on severity of disease, we made several modifications to the scoring systems from the guidelines and classified patients as 1-mild, 2-moderate, 3-severe, or 4-critical. Accordingly, taking into account the clinical symptoms and radiographic findings of the patients, the COVID-19 (+) group was classified as “mild” (individuals who had any of the signs or symptoms of COVID-19 [eg, fever, cough, sore throat, malaise, headache, muscle pain, nausea, vomiting, diarrhea, loss of taste and smell] but no shortness of breath, dyspnea, or abnormal chest imaging); “moderate” (individuals who showed evidence of lower respiratory disease during clinical assessment or imaging and who had an oxygen saturation measured by pulse oximetry [SpO_2_] of 94% or higher on room air at sea level); “severe” (individuals who had SpO_2_ <94%, respiration rate >30 breaths per minute, or >50% lung involvement on imaging); and “critical” (individuals who had respiratory failure, septic shock, and/or multiple organ dysfunction).^13^ ^,^ ^14^

Patients who had “mild” disease, were to be discharged so that their treatment would continue at home. For those who had “moderate” disease, treatment was to be at home or in hospital. For those with “severe” disease, treatment was planned as admission to the COVID-19 ward, and for those with “critical” disease, treatment was planned as admission to the COVID-19 intensive care unit (ICU). Hospital length of stay, ICU stay, requirement for mechanical ventilation, high-flow oxygen, positive inotropic support, and outcomes were recorded during hospitalization of the patients admitted to the ward or the ICU.

For the present study, based on G*Power analysis, it was determined that the COVID-19 (−) group would have 100 participants and the COVID-19 (+) group would have 280 patients. The patients in the COVID-19 (+) group were divided into four groups based on disease severity, with 70 in each group. Before the study commenced, ethical approval was received (Approval No: 2021/137).

### Biochemical Measurements

#### Blood samples

After consent for participation in the study was obtained, blood samples were collected in biochemistry tubes with separator gels and routine blood tests (complete blood count, urea, creatinine, sodium, potassium, aspartate aminotransferase, alanine transaminase, total bilirubin, lactate dehydrogenase [LDH], creatinine phosphokinase, D-dimer, ferritin, troponin, and C-reactive protein [CRP]) were performed. To measure the SCUBE-1 level, the tubes were centrifuged at 1800 × g for 10 minutes after clotting at room temperature for 20 minutes, and serum portions were carefully transferred to 1.5-milliliter (mL) capped tubes, and stored at −80°C until analyzed.

### Determination of the SCUBE-1 Level

In human sera, the SCUBE-1 level was determined using an enzyme-linked immunosorbent assay (ELISA) kit (Elabscience, Wuhan, Hubei, China; Cat No: E-EL-H5405, Lot: UPJ28DN4SW), according to the manufacturer’s recommendations.

### Transferring the Samples to ELISA Plates and Preparing for Measurements

Serum samples stored at −80°C were thawed at room temperature. The SCUBE-1 standards were prepared following the kit procedures, and 100-microliter (μL) samples were added to the wells and to the test serum samples. The plate was covered with foil and incubated on a shaker at 37°C for 90 minutes. After the liquid in the plate was removed, 100 μL of biotinylated-Ab/Ag SCUBE-1 solution was added to each well. The plate was then covered with foil and incubated on a shaker at 37°C for 60 minutes. After incubation, the liquid was removed, and the plate was washed three times with buffer using a plate washer. Then, 100 μL of streptavidin-HRP solution was added to each well. After incubation, the liquid was removed, and the plate was washed five times with buffer using a plate washer.

### Staining of Samples and Measurements

For staining, 90 μL of substrate solution was added to each well and incubated for 15 minutes in a dark environment at 37°C. Then, 50 μL of counter-staining solution was added to each well, and transformation to the color yellow was observed in each sample and the standards. The absorbance of the samples was measured at 450 nanometers on a VERSA microplate reader (Molecular Devices, LLC, San Jose, CA), and the results were recorded in nanograms (ng) per mL.

### Statistical Analysis

We used the Statistical Package for Social Sciences 23.0 (SPSS Statistics, IBM Corp, Armonk, NY) for data analysis. Categorical data are presented as numbers and percentages. The Kolmogorov-Smirnov test determined normality of the numerical data distributions. Data with a normal distribution are shown as mean ± standard deviation, and non-normally distributed data are shown with median and quartile values. We used the chi-square test in the analysis of categorical data. In the analysis of numerical data conforming to normal distribution, the Student *t*-test was used to compare two groups. In the analysis of non-normally distributed numerical data, we used the Mann-Whitney U test for two-group comparisons and the Kruskal- Wallis test with Bonferroni correction for multiple-group comparisons. The diagnostic value of the SCUBE-1 level was examined using receiver operating curve (ROC) analysis.

## RESULTS

A total of 507 patients were considered for this study. After excluding 175 patients (23 with incomplete data, two who withdrew, four with acute myocardial infarction, eight with acute stroke, 35 with acute kidney failure, one with peripheral artery disease, two with liver failure, 48 with heart failure, 48 with malignancy, two <18 years, and two pregnant patients), we completed the study with 332 patients. Among these 332 patients, 80 were COVID-19 (−) and 252 were COVID-19 (+). Among the 252 patients diagnosed with COVID-19, 74 (29.4%) were classified as mild, 95 (37.7%) as moderate, 45 (17.8%) as severe, and 38 (15.1%) as critical. Demographic and clinical characteristics, vaccination status, laboratory results, and comparisons between COVID-19 (−) and COVID-19 (+) patients are shown in Table 1.

**Table 1.** The demographic, clinical, and biochemical characteristics of COVID-19 negative and COVID-19 positive groups.

Variables	COVID-19 negative (n:80, %24.1)		COVID-19 positive (n:252, %75.9)		*P*-value
*Demographics*					
Age, years	65.0	(24.0–95.0)	63.0	(18.0–92.0)	0.73
Sex, male, n (%)	36	(45.0)	104	(41.3)	0.56
*Medical history*					
Diabetes mellitus, n (%)	20	(25.0)	55	(21.8)	0.55
Hypertension, n (%)	37	(46.3)	114	(45.2)	0.87
CVA, n (%)	7	(8.8)	12	(4.8)	0.18
CAD, n (%)	6	(7.5)	31	(12.3)	0.23
Asthma/COPD, n (%)	19	(23.8)	34	(13.5)	0.03
Smoking, n (%)	13	(16.3)	38	(15.1)	0.80
*Symptoms*					
Fever, n (%)	18	(22.5)	54	(21.4)	0.84
Cough, n (%)	28	(35.0)	165	(65.5)	<0.001
Dyspnea, n (%)	33	(41.3)	153	(60.7)	0.002
Runny nose, n (%)	5	(6.3)	7	(2.8)	0.15
Anorexia, n (%)	9	(11.3)	48	(19.0)	0.11
Loss of taste, n (%)	0	(0.0)	6	(2.4)	0.16
Loss of smell, n (%)	0	(0.0)	1	(0.4)	0.57
Myalgia, n (%)	24	(30.0)	93	(36.9)	0.26
Fatigue, n (%)	38	(47.5)	143	(56.7)	0.15
Headache, n (%)	19	(23.8)	52	(20.6)	0.55
Nausea/vomiting, n (%)	18	(22.5)	45	(17.9)	0.36
Diarrhea, n (%)	14	(17.5)	7	(2.8)	<0.001
*Vaccination,* n (%)	51	(63.7)	161	(63.9)	0.98
*Laboratory results*					
Creatinine, (mg/dL)	1.07 ± 0.4		1.09 ± 0.7		0.23
Uric acid, (mg/dL)	23.7 ± 15.7		24.5 ± 20.0		0.78
Albumin, (mg/dL)	39.5	(25.0–49.0)	39.7	(21.0–59.0)	0.06
LDH, (mg/dL)	262.5 ± 123.8		340.2 ± 163.8		0.03
CRP, (mg/dL)	74.8 ± 71.3		74.2 ± 71.6		0.05
PCT, (μg/L)	0.92 ± 2.5		2.34 ± 12.4		0.12
WBC, (×1000/mm^3^)	7.7	(3.0–20.8)	7.0	(1.2–147.0)	<0.001
Lymphocyte, (×1000/mm^3^)	1.74 ± 1.69		1.39 ± 1.93		0.009
Neutrophil, (×1000/mm^3^)	5.2	(0.4–13.6)	4.7	(12.7–118.0)	0.004
Hemoglobin, (mg/dL)	12.8	(9.8–16.3)	13.4	(5.5–18.4)	0.24
Platelet count, (×1000/mm^3^)	215.5	(123.0–417.0)	189.0	(55.0–537.0)	0.001
Fibrinogen, (mg/dL)	394.9	(201.0–735.0)	418.0	(146.0–6011.0)	0.14
D-dimer, (mg/L)	1.39 ± 1.9		1.6 ± 3.8		0.17
N/L ratio	5.9 ± 4.7		11.4 ± 27.8		0.35
Ferritin, (μg/L)	177.6 ± 164.6		621.4 ± 1022.3		<0.001

Values are expressed as mean ± SD, n (%) or median (interquartile range) unless otherwise stated.

*CAD*, coronary artery disease; *COPD*, chronic obstructive pulmonary disease; *CRP*, C-reactive protein; *CVA*, cerebrovascular accident; *LDH*, lactate dehydrogenase; *N/L* ratio, neutrophil/lymphocyte ratio; *PCT*, procalcitonin; *WBC*, white blood cell.

**Figure 1.**
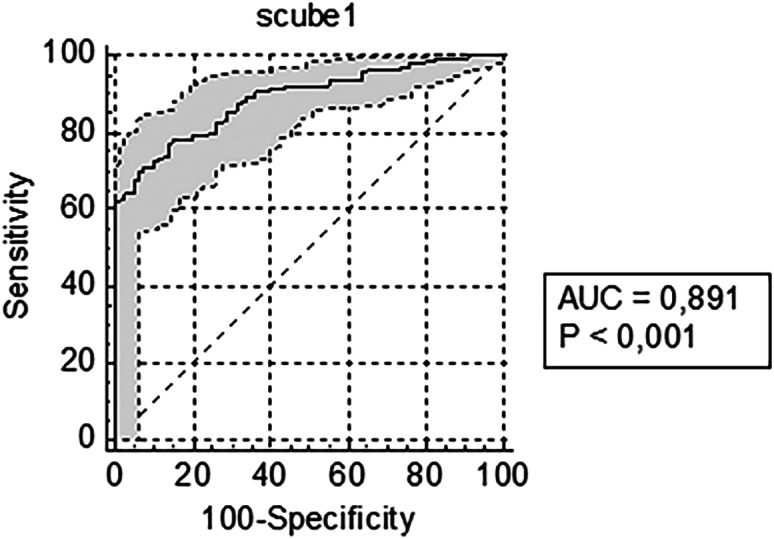
The initial SCUBE-1* level cut-off and confidence interval values for COVID-19 diagnosis. *SCUBE-1, signal peptide-CUB-EGF domain-containing protein 1. *AUC*, area under the curve; *NPV*, negative predictive value.

**Figure 2.**
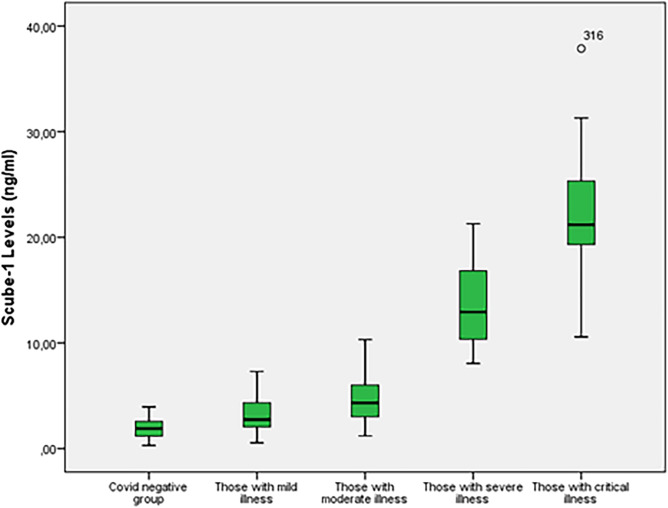
*SCUBE-1 levels of COVID-19 patients. All *P*-values <0.001. *SCUBE-1, signal peptide-CUB-EGF domain-containing protein 1.

The comparison of the SCUBE-1 level between the COVID-19 (−) and COVID-19 (+) groups is presented in Table 2. There was a significant difference in the diagnostic value of SCUBE-1 for COVID-19 between the COVID-19 (−) and COVID-19 (+) groups (*P* < 0.001). The COVID-19 (+) patients (8.48 ± 7.42) had a higher mean SCUBE-1 level than COVID-19 (−) patients (1.86 ± 0.92) (Table 2). The ROC analysis and initial SCUBE-1 level cut-off values for COVID-19 diagnosis (area under the curve [AUC] 0.891, confidence interval [CI] 0.852–0.922, *P* < 0.001) are shown in Figure 1 and Table 3. For the COVID-19 (+) group (n = 252) the SCUBE-1 level increased with an increase in disease severity (*P* < 0.001). Comparisons between SCUBE-1 levels for COVID-19 (+) patients, classified according to disease severity, are shown in Table 2 and Figure 2.

**Table 2.** The SCUBE-1^*^ levels of COVID-19 negative and COVID-19 positive groups according to disease severity and patient outcomes.

		SCUBE-1 levels (ng/mL)	*P-*value
	COVID-19 (−), (n = 80)	1.86 ± 0.92	<0.001^*^
	COVID-19 (+), (n = 252)	8.48 ± 7.42	
COVID-19 (+) (n = 252)	Mild, (n = 74)	3.20 ± 1.65	<0.001^¥^
	Moderate, (n = 95)	4.78 ± 2.26	
	Severe, (n = 45)	13.68 ± 3.95	
	Critical, (n = 38)	21.87 ± 5.39	
COVID-19 (+) (n = 252)	Discharged, (n = 94)	2.89 (0.55–8.60)	<0.001^¥^
	Ward-admitted, (n = 120)	7.13 (1.38–21.29)	
	ICU-admitted, (n = 38)	21.19 (10.58–37.86)	

* Mann-Whitney U test.

¥ Kruskal-Wallis test with Mann-Whitney correction, results achieved from the comparison of the three groups were statistically significant.

* SCUBE-1, signal peptide-CUB-EGF domain-containing protein 1.

**Table 3.** SCUBE-1^*^ cut-off value of COVID-19 negative and COVID-19 positive groups.

	Sensitivity		Specificity		PPV		NPV	
SCUBE-1 cut-off value (ng/mL)	(%)	(95% CI)	(%)	(95% CI)	(%)	(95% CI)	(%)	(95% CI)
0.54	99.6	(97.8–100.0)	8.7	(3.6–17.2)	77.5	(76.3–78.7)	87.5	(46.6–98.2)
2.05	90.8	(86.6–94.1)	63.7	(52.2–74.2)	88.8	(85.5–91.4)	68.9	(59.2–77.2)
3.89	62.3	(56.0–68.3)	98.7	(93.2–100.0)	99.4	(95.7–99.9)	45.4	(41.5–49.4)

* SCUBE-1, signal peptide-CUB-EGF domain-containing protein 1.

*CI*, confidence interval; *NPV*, negative predictive value; *PPV*, positive predictive value.

**Figure 3.**
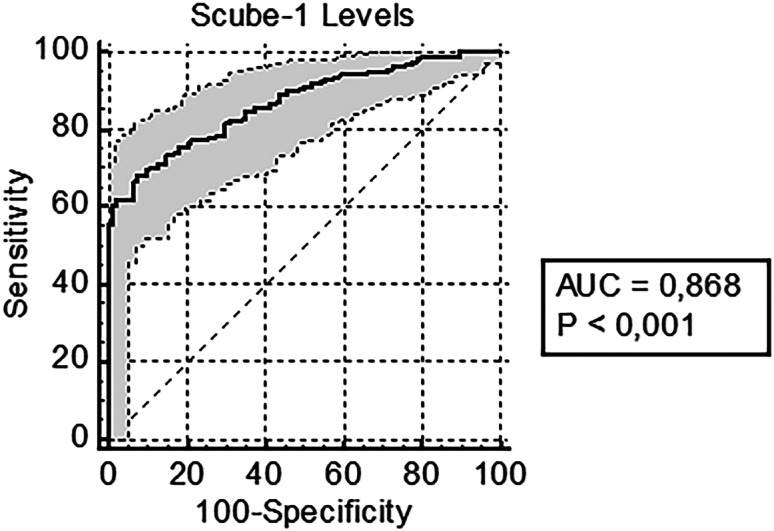
The cut-off and confidence interval values for the safe discharge of COVID-19 (+) patients according to ROC analysis. *ROC*, receiver operating characteristic curve; *SCUBE-1*, signal peptide-CUB-EGF domain-containing protein 1.

Among the 252 patients in the COVID-19 (+) group, 118 were admitted to the COVID-19 ward and 38 to the COVID-19 ICU, in line with current guidelines and the protocol published by the Republic of Türkiye Ministry of Health. Ninety-six patients, who were COVID-19 (+) and did not require hospitalization based on the clinical and laboratory examinations, were discharged. Thirteen of the 96 discharged patients (13.5%) returned to the ED within 14 days after discharge, but only two of these 13 were hospitalized. These two patients were added to the hospitalized patient group for the statistical analysis. Therefore, although 94 patients were discharged, 120 patients were considered to be hospitalized because of COVID-19. The mean SCUBE-1 level of the discharged patients was significantly lower than that of patients requiring hospitalization (*P* < 0.001). The COVID-19 (+) patients were divided into three groups based on their outcomes: discharged; admitted to the ward; and admitted to the ICU. The SCUBE-1 levels and comparisons by patient outcomes are presented in Table 2. The cut-off values for the safe discharge of patients were determined with ROC analysis (AUC 0.868, CI 0.820–0.907, *P* < 0.001) (Figure 3). The ROC curves and optimal cut-off values are shown in Table 4 and Figure 3.

**Table 4.** Optimal SCUBE-1^*^ cut-off values of COVID-19 positive patients according to patients requiring hospitalization outcomes.

Patients requiring hospitalization outcome	SCUBE-1 cut-off value (ng/mL)	Sensitivity		Specificity		PPV		NPV	
		(%)	(95% CI)	(%)	(95% CI)	(%)	(95% CI)	(%)	(95% CI)
Discharged	1.38	99.3	(96.5–100.0)	10.6	(5.2–18.7)	65.1	(63.5–66.7)	90.9	(56.5–98.7)
Ward admitted	3.05	89.8	(84.1–94.1)	54.2	(43.7–64.6)	76.8	(72.5–80.5)	76.1	(65.9–84.0)
ICU admitted	8.27	55.7	(47.6–63.6)	98.9	(94.2–100.0)	98.9	(92.6–99.8)	57.1	(52.7–61.3)

* SCUBE-1, signal peptide-CUB-EGF domain-containing protein 1.

*CI*, confidence interval; *ICU*, intensive care unit; *NPV*, negative predictive value; *PPV*, positive predictive value.

## DISCUSSION

The results from this study show that the mean SCUBE-1 level of COVID-19 (+) patients was higher than for COVID-19 (−) patients and that the SCUBE-1 level increases with severity of the disease. Moreover, there was a significant difference between patients requiring hospitalization outcomes, such as discharged, admitted to the ward, admitted to the ICU, and the SCUBE-1 level.

Many studies have examined the diagnostic and prognostic effectiveness of reverse transcription-polymerase chain reaction (RT-PCR) using immunological, biochemical, and hematological parameters in patients with COVID-19, and differences between studies have been reported. Although RT-PCR is known as the gold standard diagnostic method to diagnose COVID-19, it has some drawbacks,^15^ ^,^ ^16^ such as incorrect sample collection and low viral loads, which may cause false negative results.^15^ Another study reported that 3% of patients presenting with COVID-19 symptoms had COVID-19-related tomography findings; however, while RT-PCR test results for these patients were negative, serial RT-PCR tests during follow-up of these patients were positive.^17^ Considering that the average six-day incubation period of the severe acute respiratory coronavirus 2 (SARS-CoV2), RT-PCR results in the early symptomatic period or during the recovery period may yield negative results.^18^ In both cases, it may become difficult to control the infection and prevent a pandemic because contagiousness continues, which may cause delayed treatment in patients.^19^ Moreover, the diagnosis requires a longer period, and serial testing is expensive. Therefore, simpler diagnostic tests are required in resource-limited regions where RT-PCR cyclers and highly trained technicians are not employed.

The effectiveness of IgM and IgG antibodies detection in the diagnosis of COVID-19 has been investigated previously, and it was shown in serology-based tests that sensitivity increased as the time from symptom onset increased, and sensitivity was relatively lower before seven days.^20^ ^–^ ^23^ Furthermore, the diagnostic value of biochemical and hematological biomarkers (eg, D-dimer, CRP, procalcitonin, LDH, ferritin, lymphocyte count, and leukocyte count) was examined for COVID-19, and these biomarkers were shown to have low diagnostic efficiency and higher prognostic value than diagnostic value.^24^ ^–^ ^31^ The data obtained suggests that low-cost, easy-to-obtain, and easy-to-use biomarkers that provide results in a short time and offer high diagnostic efficiency are required.

The SCUBE-1 is highly expressed in vascular endothelial cells and platelets and is known to increase in thrombotic diseases with platelet and endothelial activation.^32^ ^,^ ^33^ Therefore, SCUBE-1 has been used for diagnostic or prognostic evaluation of many thrombotic diseases. In a previous study, the SCUBE-1 level in patients diagnosed with pulmonary embolism was higher than in the control group, and it was stated that the SCUBE-1 level could be used for the diagnosis of pulmonary embolism at a cut-off point of >46 ng/mL with 82% sensitivity and 91% specificity.^34^ Similarly, Xiao et al reported that SCUBE-1 may be a potential biomarker for the diagnosis of pulmonary embolism.^35^ Cakir et al determined the diagnostic value of SCUBE-1 in aortic dissection and reported that it could be used for the diagnosis of aortic dissection in patients with aortic dissection at levels >19.75 ng per deciliter with 95% sensitivity and 76% specificity.^36^ Furthermore, Dai et al concluded in their study that the SCUBE-1 level may have diagnostic value in patients with acute coronary syndrome and acute ischemic stroke.^37^

Studies that examined the diagnostic effectiveness of SCUBE-1 were based on the relationship between SCUBE-1 and thrombus formation due to endothelial dysfunction. SARS-CoV-2 does not possess procoagulant characteristics,^32^ ^,^ ^38^ but vascular endothelial cell damage occurs due to an excessive inflammatory response triggered by COVID-19. The diagnostic relationship between SCUBE-1 and COVID-19 was determined in the present study because hypercoagulability, platelet activation, and endothelial dysfunction may develop with the resulting vasculopathy.^34^ ^,^ ^39^ ^,^ ^40^ One of the main results of this study was that the SCUBE-1 level was higher in COVID-19 (+) patients than in COVID-19 (−) patients. In this context it showed that the SCUBE-1 level is an effective biomarker for the diagnosis of COVID-19, and it can be used to diagnose COVID-19 in EDs. However, the current assay studied here is complicated and labor intensive and would take at least 210 minutes to perform, even under optimum conditions.^41^

Clinical signs and symptoms (eg, cough, dyspnea, fever, diarrhea, nausea, vomiting, loss of taste and smell, respiratory rate, saturation, and radiographic findings) were used to determine the severity of COVID-19.^12^ Many biochemical parameters (eg, elevated CRP, thrombocytopenia, and an elevated ferritin level) are poor prognostic factors in COVID-19, and they have not been used to define disease severity per the current literature.^42^ ^–^ ^44^ In this context, determining the disease severity of patients at the time of admission by using a biochemical parameter, such as the SCUBE-1 level (with or without the present scoring systems), can be used at an early stage to distinguish between severe and critical patients with COVID-19 to reduce mortality and enable timely treatment. Microvascular and macrovascular thrombotic complications may develop in arterial, venous, and capillary vascular beds because thromboinflammatory processes intensify during COVID-19, particularly with increasing severity of the disease.^39^ ^,^ ^45^ In a 2022 study conducted by Toprak et al, an elevated SCUBE-1 level was associated with thrombotic complications, disease severity, and inhospital mortality in patients with COVID-19.^46^ In the present study, the SCUBE-1 level was elevated in patients with COVID-19, and as the severity of the disease increased the SCUBE-1 level also increased.

The study conducted by Calik et al reported a low mortality rate of patients who presented early to hospital and received early antiviral treatment.^47^ Early diagnosis, appropriate triage, and early treatment of patients who present to healthcare institutions with symptoms of COVID-19 and are considered COVID-19 (+) may prevent the risk of contamination, reduce the need for intensive care, and reduce the need for hospitalization by enabling rapid decision-making in the best interests of patients. From this perspective, biomarkers are required to guide clinicians in hospitalization/discharge decisions and ward/intensive care unit admission of patients diagnosed with COVID-19.^48^ ^,^ ^49^ Such a biomarker may contribute to better decision-making at the ED or discharge stage and ED occupancy by reducing patient wait times.

In the present study, when the ED outcomes of the patients were grouped as discharge, ward admission, or intensive care admission, and when the SCUBE-1 levels were compared, the SCUBE-1 level of discharged patients was lower than that of patients who required hospitalization, and the SCUBE-1 level of patients who required ICU admission was higher than for the other groups. Given these results, it can be argued that the SCUBE-1 level may assist clinicians to predict disease severity and assist in making decisions regarding hospitalization or discharge. In addition, because of the risk of micro- and macrovascular thrombosis, a high SCUBE-1 level measured in the early stages of the disease may indicate the requirement for more intensive antithrombotic treatment to prevent thrombotic complications.

The RT-PCR is the gold standard for confirming the presence of SARS-CoV2, and the time to obtain the result for a single test is approximately two hours.^50^ However, samples collected in hospitals were transported to specific laboratories because PCR tests could not be performed in every laboratory during the pandemic period,^51^ ^,^ ^52^ which resulted in delays in receiving the test results. Previous studies have shown that the confirmation time of the SARS-CoV-2 virus using RT-PCR was 6–48 hours during the pandemic period.^53^ ^,^ ^54^ In addition, tests such as the RT-PCR only identify SARS-CoV2 and do not provide data on the severity of COVID-19. Considering these limitations, the use of RT-PCR kits for surveillance or screening patients, preventing increased patient density in healthcare institutions, and reducing patient wait times may be difficult.^53^ Therefore, there is a need for novel biomarkers to enable the rapid detection of individuals with COVID-19, even in primary healthcare institutions and to guide physicians regarding the discharge or hospitalization of patients according to cut-off values.

The test time of SCUBE-1 is approximately 3.5 hours, and the sample is easy to obtain from a blood sample, which enables the rapid identification of patients with COVID-19.^41^ The present study also revealed that the SCUBE-1 level is associated with the severity of disease, which facilitates decision-making regarding discharge or admission to the ward or the ICU, which may assist in reducing patient density in healthcare institutions, reduce patient wait times, and effectively improve patient management.

## LIMITATIONS

There were some limitations to this study. First, the targeted number of patients was not recruited owing to the decreased severity and incidence of COVID-19 worldwide. Second, because the number of SCUBE-1 kits was limited, SCUBE-1 measurements were limited to a single plasma sample. Serial SCUBE-1 measurements during patient treatment may have altered the correlation between the SCUBE-1 level and disease severity.

## CONCLUSION

Even though RT-PCR testing usually produces a diagnosis of COVID-19 in a short time, the excessive sample load accumulated in laboratories during the pandemic increased the time to completion and increased patient wait times. In the present study, we found that the SCUBE-1 level differs between patients with and without COVID-19 and it was correlated with the severity of the disease. Accordingly, besides guiding physicians regarding the diagnosis of COVID-19 and the severity of the disease among patients who present at health facilities during pandemic periods where results of RT-PCR tests may be delayed, SCUBE-1 may assist clinicians in managing inflammatory diseases that predispose to thrombosis.
